# Dose escalation in oropharyngeal cancer: a comparison of simultaneous integrated boost and brachytherapy boost

**DOI:** 10.1186/s13014-023-02256-x

**Published:** 2023-04-07

**Authors:** Anna Embring, Eva Onjukka, Claes Mercke, Ingmar Lax, Anders Berglund, Signe Friesland

**Affiliations:** 1 Department of Oncology-Pathology, Karolinska Institutet, Stockholm, Sweden; 2 Department of Oncology, Karolinska University Hospital, Anna Steckséns Gata 41, 171 76 Solna, Stockholm, Sweden; 3 Medical Radiation Physics and Nuclear Medicine, Karolinska University Hospital, Stockholm, Sweden; 4 Epistat Epidemiology and Statistics Consulting, Uppsala, Sweden

**Keywords:** Radiotherapy, Simultaneous integrated boost, Brachytherapy, Dose escalation, Oropharyngeal cancer, Head and neck cancer, Side effects

## Abstract

**Background:**

Local recurrence is the most common pattern of failure in head and neck cancer. It can therefore be hypothesised that some of these patients would benefit from an intensified local treatment, such as radiation dose escalation of the primary tumour. This study compares treatment and toxicity outcomes from two different boost modalities in oropharyngeal cancer: simultaneous integrated boost (SIB) and brachytherapy boost.

**Methods:**

Two hundred and forty-four consecutive patients treated with > 72 Gy for oropharyngeal squamous cell carcinoma between 2011 and 2018 at our institution were retrospectively analysed. Data on side effects were collected from a local quality registry and supplemented with a review of medical records. Patients receiving a brachytherapy boost first had external beam radiotherapy consisting of 68 Gy in 2 Gy fractions to the gross tumour volume (GTV), and elective radiotherapy to the neck bilaterally. The brachytherapy boost was typically given using pulsed dose rate, 15 fractions and 0.56–0.66 Gy per fraction [total dose in EQD2 = 75.4–76.8 Gy (α/β = 10)]. The typical dose escalated radiotherapy with external beam radiotherapy only, was delivered using SIB with 74,8 Gy in 2.2 Gy fractions [EQD2 = 76.0 Gy (α/β = 10)] to the primary tumour, 68 Gy in 2 Gy fractions to GTV + 10 mm margin and elective radiotherapy to the neck bilaterally.

**Results:**

Dose escalation by SIB was given to 111 patients and brachytherapy boost to 134 patients. The most common type of cancer was base of tongue (55%), followed by tonsillar cancer (42%). The majority of patients had T3- or T4-tumours and 84% were HPV-positive. The 5-year OS was 72,4% (95% CI 66.9–78.3) and the median follow-up was 6.1 years. Comparing the two different dose escalation modalities we found no significant differences in OS or PFS and these results remained after a propensity-score matched analysis was performed. The analysis of grade ≥ 3 side effects showed no significant differences between the two different dose escalation techniques.

**Conclusions:**

We found no significant differences in survival or grade ≥ 3 side effects comparing simultaneous integrated boost and brachytherapy boost as alternative dose escalation modalities in the treatment of oropharyngeal cancer.

**Supplementary Information:**

The online version contains supplementary material available at 10.1186/s13014-023-02256-x.

## Background

In head and neck cancer (HNC) the most common mode of recurrence is local failure [1, 2]. It can therefore be hypothesised that some subgroups of HNC patients would benefit from an intensified local treatment, such as radiation dose escalation. Altered fractionation schedules and the possibilities of dose escalation have been explored since the mid-eighties, with the intent to achieve increased local control with acceptable side effects [3, 4]. Technical advances, for example the development of intensity modulated radiotherapy (IMRT), have made it possible to achieve more conformal dose distributions which offer the potential to deliver higher target doses without necessarily giving higher doses to organs at risk. Several studies have shown that dose escalation in HNC can be achieved with acceptable side effects [5–8]. In our institution, no significant increase in serious side effects compared to standard dose fractionation was found at an interim analysis in 2015. The current study uses the definition of escalated versus standard dose from the National Comprehensive Cancer Network Guidelines [9] of > 72 Gy, which has also been used in previous studies [7].

A frequently used dose-escalation technique is simultaneous integrated boost (SIB). This technique, using IMRT or Volumetric Modulated Arc Therapy (VMAT), where an inhomogeneous dose distribution in every fraction will enable dose escalation in selected volumes, has been used for the last two decades [10–12] and SIB is now a commonly used technique, not only in dose escalation but also in achieving standard dose treatment with lower doses in elective volumes. Another technique to achieve dose escalation is boosting a selected volume with brachytherapy. This technique predates SIB and has been used in HNC for over half a century [13, 14]. Important pioneer work on brachytherapy in HNC was done in France and they developed the technique to become a useful treatment modality [15]. Brachytherapy offers a unique possibility to achieve local dose escalation in tumours with a rapid dose fall-off to surrounding healthy tissues, thus serving as an effective boost therapy, and is often used in combination with external beam radiotherapy [13, 15]. A commonly used schedule in HNC is external beam radiotherapy to approximately 50 Gy and a subsequent brachytherapy boost of an additional 20–30 Gy [15–18]. The treatment in our clinic differs from this in that the patients first receive external beam radiotherapy to a standard dose of 68 Gy, and then a brachytherapy boost to the primary tumour of 8.4 Gy in 15 fractions (current practice). This treatment schedule was established to enable adequate doses to any lymph node metastases of the neck before the brachytherapy boost to the primary tumour. The two different boost modalities explored in this study are well described in the literature, and the SIB technique is widely available. The technique of using brachytherapy in the head and neck area is more complex and requires special equipment and trained staff but is used at many centres around the world. However, comparisons of treatment outcome from these boost modalities for dose escalation in HNC are scarce. Chen et al. reports their single centre experience in treating base of tongue cancer and briefly compares the two techniques, but not in the dose escalation setting [19].

The aim of this study is to compare clinical outcomes and side effects of two different modalities of dose escalated radiotherapy with equivalent target dose, SIB, and brachytherapy boost, in oropharyngeal cancer.

## Methods

### Patients

Two hundred and forty-four consecutive patients treated with > 72 Gy (EQD2 α/β = 10 Gy) for oropharyngeal squamous cell carcinoma between 2011 and 2018 at our institution were included in the analysis. Patients were mainly identified through our local quality registry, but to avoid selection bias (patients are registered in the local quality registry at their follow-up visits after completing radiotherapy) a manual search in our treatment planning system was performed to identify all eligible patients, including patients who failed to attend follow-up visits.

Pre-treatment evaluations included complete medical history, physical examination including panendoscopy and diagnostic contrast enhanced computed tomography (CT). The diagnostic imaging sometimes also included positron emission tomography (PET) CT or magnetic resonance imaging (MRI) at the clinician´s discretion. All patients were discussed at a multidisciplinary tumour board before starting treatment, and staged according to the American Joint Committee on Cancer’s (AJCC) Cancer-Staging Manual, 7th edition [20]. The decision to treat with dose escalated radiotherapy was made by the treating physician based on local guidelines, where criteria for qualifying were specified. Primarily oropharyngeal cancer with large primary tumours (T3–T4 tumours) were selected for dose escalated radiotherapy, but sometimes also smaller primary tumours located in the base of tongue as they have poorer prognosis compared to tonsillar cancer with corresponding T-stage [21, 22]

The study was approved by the National Ethical Review Authority.

### Treatment

#### External beam radiotherapy

**Fig. 1:**
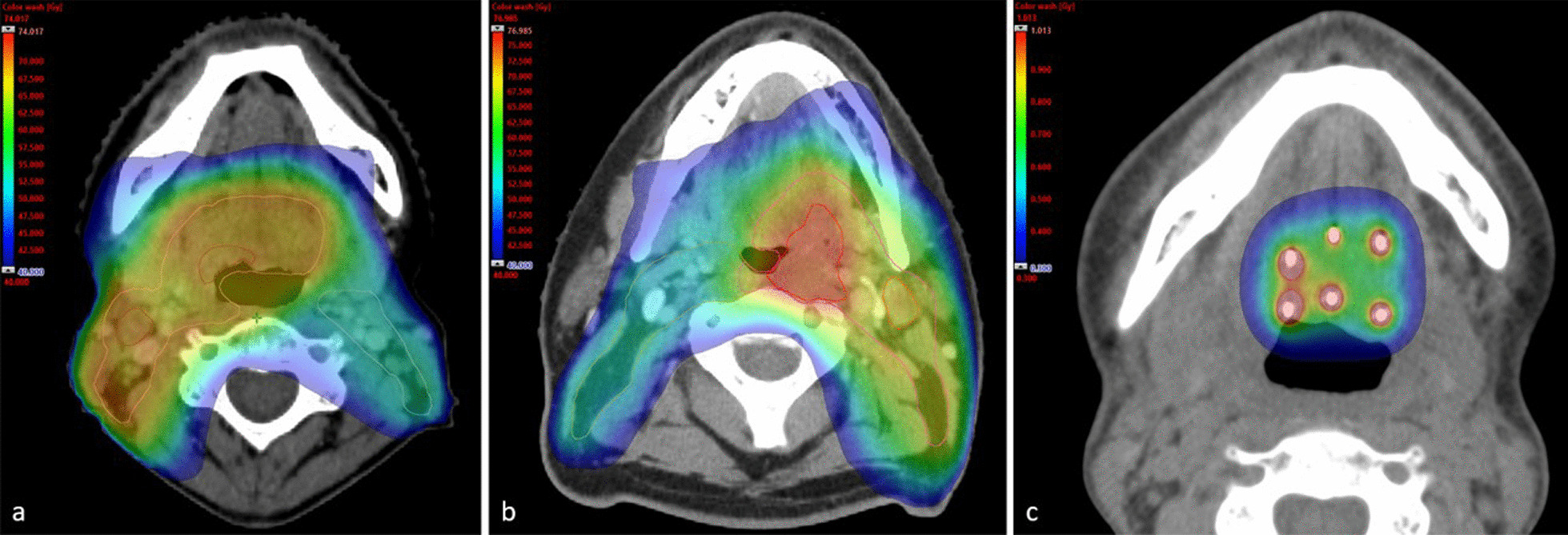
Pictures of dose distribution in colour wash. **a** Picture with an example of external beam radiotherapy with standard dose to high-risk volumes and contralateral elective lymph node irradiation. **b** Picture with an example of a simultaneous integrated boost with dose escalation to the primary tumour, standard dose to high-risk volumes and contralateral elective lymph node irradiation. **c** Picture with an example of a brachytherapy boost

During external beam radiotherapy, patients were immobilized by a moulded 5-point mask and treated in a supine position. A CT scan with 2–2.5 mm slice thickness was used for treatment planning, including intravenous contrast unless contraindicated. Standard treatment was 68 Gy in 2 Gy fractions to the gross tumour volume (GTV) with a 10 mm margin and elective radiotherapy to the neck bilaterally with either 51.68 Gy using 1.52 Gy per fraction [EQD2 = 49,6 Gy (α/β = 10)], when SIB was used, or 46 Gy in 2 Gy fractions, when a sequential boost was used (Fig. 1a). Elective lymph node irradiation routinely included levels II-IV bilaterally. A planning target volume (PTV) was created by an isotropic 5 mm margin for the standard- and elective dose targets, and an isotropic 3 mm margin for the dose-escalated target. The standard technique was VMAT using 6 MV photons. The external beam radiotherapy was planned in Eclipse (Varian, USA).

The standard dose escalated radiotherapy with external beam radiotherapy only (applying to 90% of these patients), was delivered using SIB with 74.8 Gy in 2.2 Gy fractions [EQD2 = 76.0 Gy (α/β = 10)] to the primary tumour (GTVT) with a 0–5 mm margin, 68 Gy in 2 Gy fractions to GTV + 10 mm margin and elective radiotherapy to the neck bilaterally with 51.68 Gy using 1.52 Gy per fraction (Fig. 1b). The outlines of this treatment are well described in a local protocol. The remaining 10% of patients in this group were treated with slightly different fractionation schedules, mainly because they were included in other dose escalation studies and were treated according to separate study protocols. A detailed list of all fractionation schedules used is supplied in the Additional file 1 (Table S1).

#### Brachytherapy

All patients in this study who were treated with brachytherapy received external beam radiotherapy to 68 Gy (as described above) followed by brachytherapy, which was given as a boost to the primary tumour with a safety margin of 5–10 mm, approximately one week after completion of the external beam radiotherapy. Implantation of catheters was done under general anaesthesia in an operating theatre by a radiation oncologist specialized in head and neck brachytherapy in collaboration with an ear, nose and throat surgeon. The technique used is the same as in Centre Alexis Vautrin in Nancy, France, described in an article by Pernot et al. [23]. The typical treatment (94% of patients receiving a brachytherapy boost) was a brachytherapy boost using pulsed dose rate (PDR) consisting of 15 fractions, 0.56–0.66 Gy per fraction [total dose in EQD2 = 75.4–76.8 Gy (α/β = 10)], delivered every hour during office hours, over a total time of 2–3 days (Fig. 1c). Seven patients (5%) received high dose rate brachytherapy (HDR) by clinician’s choice. All brachytherapy was delivered using an afterloading device and treatments were planned in Oncentra (Elekta, Sweden). A detailed list of all fractionation schedules used is supplied in the Additional file 1 (Table S1).

All doses reported in this study are prescribed doses.

### Systemic medical treatment

Standard concurrent medical treatment was either weekly cisplatin (40 mg/m^2^ once a week during radiotherapy, maximum dose of 70 mg) or weekly cetuximab (400 mg/m^2^ one week before start of radiotherapy and thereafter 250 mg/m^2^ weekly during radiotherapy) in both groups. When induction chemotherapy was applied, the patient was treated with a combination of docetaxel (75 mg/m^2^, maximum dose of 150 mg), cisplatin (75 mg/m^2^, maximum dose of 150 mg) and fluorouracil (1000 mg/m^2^/24 h by continuous infusion over 4 days, maximum dose of 2000 mg/24 h), administered every 21 days for 2 cycles before start of radiotherapy.

### Toxicity outcomes

Side effect data were collected from the local quality registry and supplemented with a review of medical records. Side effects occurring during or within 90 days of the end of radiotherapy were considered acute side effects, and side effects occurring later were considered late side effects. The local quality registry includes prospectively gathered toxicity data on all patients treated for HNC with curative intent in our institution. Data are collected during patients’ follow-up visits, every three months the first two years, and then every six months for another three years. Toxicities (acute and late) recorded are: skin-, mucosa- and larynx toxicity and trismus, and late toxicities only: salivary gland toxicity, dysphagia, and osteoradionecrosis (ORN). Grading of ORN was according to Late effects Normal Tissue Task Force Subjective, Objective, Management, and Analytic (LENT/SOMA) scores [24]. All other side effects were graded according to Radiation Therapy Oncology Group (RTOG) and the European Organization for Research and Treatment of Cancer (EORTC) [25]. Side effects were considered severe at grade ≥ 3. Closure of database was 17th of September 2021.

### Statistics

Clinical characteristics by boost modality was presented using descriptive statistics and tested by Chi-square tests for categorical data, and Wilcoxon tests for continuous variables. The Kaplan–Meier approach was used to estimate overall survival (OS) and progression-free survival (PFS). OS was defined as the time from the last day of radiotherapy to death or last date of follow up, whichever came first. PFS was defined as the time from the last day of radiotherapy to progression, death, or last date of follow up, whichever came first. In a first step, OS and PFS was presented for the full cohort by boost modality. In a subsequent step, propensity score matching was used to match the boost modalities by human papillomavirus (HPV) status, age, stage, performance status, gender, concurrent medical treatment, induction chemotherapy and medical treatment (concurrent or induction) using the Nearest Neighbour method with a caliper level of 0.1. Statistical significance was set to 5% and the statistical analyses were performed using R version 4.1.2.

## Results

Two hundred and forty-four patients with oropharyngeal cancer were included in this study and the median follow-up time was 6.1 years (interquartile range 3,7–8,0) in all patients. Dose escalation by SIB was given to 111 patients and brachytherapy boost to 134 patients. One patient had both dose-escalated SIB to 74.8 Gy and then a subsequent brachytherapy boost of 8.4 Gy (due to palpable residual tumour at end of external beam radiotherapy). This patient is included in the overall analysis of the dose-escalated cohort (OS and PFS) but is excluded from analyses comparing dose-escalated SIB and brachytherapy boost. The median age at start of radiotherapy was 62 years (range 31–80) and the most common type of cancer was base of tongue (55%), followed by tonsillar cancer (42%). There was a predominance of advanced stage primary tumours with 57% being T3 or T4 tumours and the majority of patients were HPV positive (84%). In the cohort treated with brachytherapy boost, the most common tumour site was the base of tongue (69%) while, in contrast, the most common tumour site in the SIB cohort was tonsil (58%). There was also a predominance of more advanced T stages in the SIB cohort compared to the cohort receiving brachytherapy boost. For further details on baseline characteristics, see Table 1. Diagnostic imaging before target delineation was CT only in 78% of patients, MRI in addition to CT in 18%, PET in addition to CT in 2% CT, and MRI and PET in addition to CT in 2%.

**Table 1:** Clinical characteristics of full cohort and the two treatment groups

Clinical characteristics	Overall	SIB	Brachy	*p* value
	Number (%)			
All subjects	243	110	133	
HPV-status				0.610
Negative	34 (14.0)	14 (12.7)	20 (15.0)	
Positive	205 (84.4)	95 (86.4)	110 (82.7)	
Unknown	4 (1.6)	1 (0.9)	3 (2.3)	
Age				0.223
> Median (62 years)	122 (50.2)	50 (45.5)	72 (54.1)	
≤ Median (62 years)	121 (49.8)	60 (54.5)	61 (45.9)	
Tumour stage*				0.389
I-II	17 (7.0)	5 (4.5)	12 (9.0)	
III	29 (11.9)	13 (11.8)	16 (12.0)	
IV	197 (81.1)	92 (83.6)	105 (78.9)	
T stage				< 0.001
1	32 (13.2)	3 (2.7)	29 (21.8)	
2	72 (29.6)	28 (25.5)	44 (33.1)	
3	71 (29.2)	39 (35.5)	32 (24.1)	
4	68 (28.0)	40 (36.4)	28 (21.1)	
Tumour site				< 0.001
Base of tongue	134 (55.1)	42 (38.2)	92 (69.2)	
Tonsil	102 (42.0)	64 (58.2)	38 (28.6)	
Other†	7 (2.9)	4 (3.6)	3 (2.3)	
Performance status (PS)				0.359
PS 0	208 (85.6)	96 (87.3)	112 (84.2)	
PS 1	29 (11.9)	13 (11.8)	16 (12.0)	
PS 2	6 (2.5)	1 (0.9)	5 (3.8)	
Gender				0.963
Male	187 (77.0)	84 (76.4)	103 (77.4)	
Female	56 (23.0)	26 (23.6)	30 (22.6)	
Concurrent medical treatment				0.042
Cetuximab	111 (45.7)	51 (46.4)	60 (45.1)	
Cetuximab + Cisplatin	9 (3.7)	5 (4.5)	4 (3.0)	
Cisplatin	74 (30.5)	40 (36.4)	34 (25.6)	
None	49 (20.2)	14 (12.7)	35 (26.3)	
Induction chemotherapy				< 0.001
Yes	93 (38.3)	27 (24.5)	66 (49.6)	
No	150 (61.7)	83 (75.5)	67 (50.4)	
Medical treatment (concurrent or induction)				0.944
Yes	215 (88.5)	98 (90.1)	117 (88.0)	
No	28 (11.5)	12 (10.9)	16 (12.0)	
Cisplatin containing medical treatment				0.069
Yes	151 (62.1)	61 (55.5)	90 (67.7)	
No	92 (37.9)	49 (44.5)	43 (32.3)	
Smoking status at start of radiotherapy				0.861
Never	84 (34.6)	40 (36.4)	44 (33.1)	
Current	53 (21.8)	23 (20.9)	30 (22.6)	
Former	106 (43.6)	47 (42.7)	59 (44.4)	
Recurrence				0.212
No recurrence	193 (79.4)	87 (79.1)	106 (79.7)	
Local recurrence	25 (10.3)	11 (10.0)	14 (10.5)	
Regional recurrence	4 (1.6)	0 (0.0)	4 (3.0)	
Distant metastasis	10 (4.1)	5 (4.5)	5 (3.8)	
Distant metastasis + local/regional recurrence	8 (3.3)	4 (3.6)	4 (3.0)	
Cannot be assessed	3 (1.2)	3 (2.7)	0 (0.0)	

*P* value < 0.05 indicates significant differences in the distribution of clinical characteristics between the two treatment groups

*SIB* Simultaneous integrated boost, *Brachy* Brachytherapy boost in combination with external beam radiotherapy, *HPV* Human papillomavirus, *PS* Performance status according to WHO

*Tumour stage according to the American Joint Committee on Cancer’s (AJCC) Cancer-Staging Manual, 7th edition

^†^3 patients with soft palate cancer and 4 patients with oropharyngeal cancer not otherwise specified

**Fig. 2:**
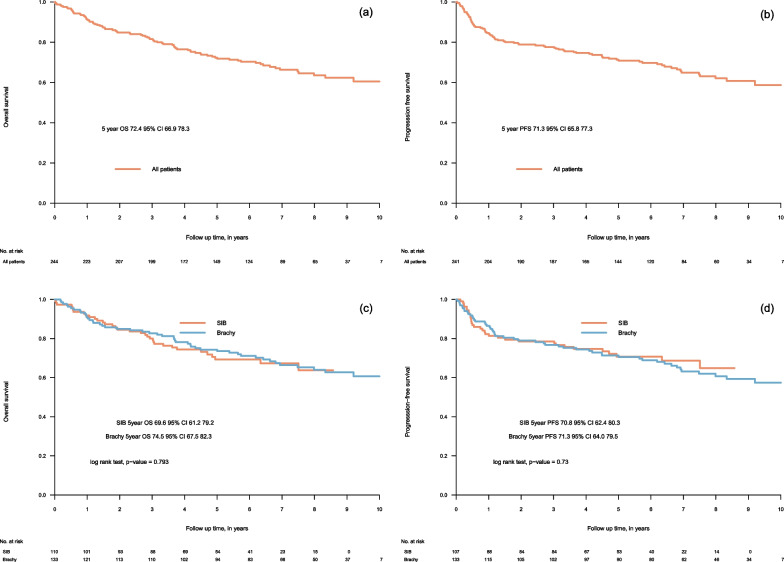
Overall survival in total cohort (**a**), progression-free survival in total cohort (**b**), overall survival by boost modality (**c**) and progression-free survival by boost modality (**d**). SIB—simultaneous integrated boost, Brachy—brachytherapy boost in combination with external beam radiotherapy

**Fig. 3:**
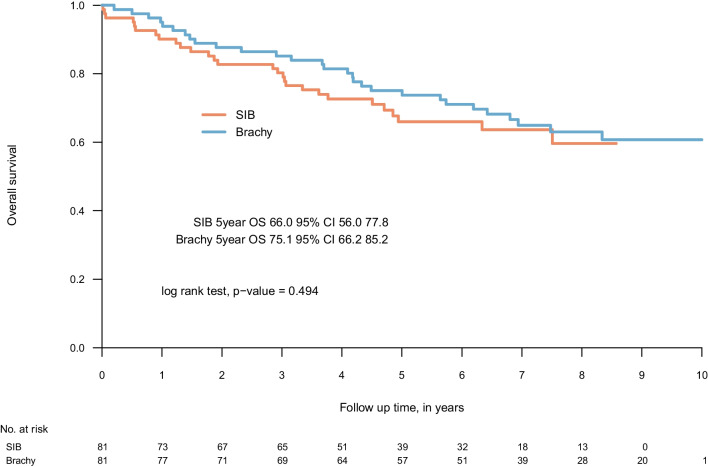
Propensity score matched overall survival by boost modality. SIB—simultaneous integrated boost, Brachy—brachytherapy boost in combination with external beam radiotherapy

The 5-year OS and PFS was 72.4% (95% CI 66.9–78.3) and 71.3% (95% CI 65.8–77.3) respectively, in the whole cohort (Fig. 2a, b). Comparing the two different dose escalation modalities we found no significant differences in OS or PFS (Fig. 2c, d). Similarly, no differences were found in a propensity score matched analysis, compensating for a slight imbalance in clinical characteristics (Table 2, Fig. 3, and Additional file 1: Fig. S1). Further, we found no statistically significant differences in OS or PFS according to gender or primary tumour site (see Additional file 1: Fig S2).

**Table 2:** Clinical characteristics by boost modality (propensity score matched)

Clinical characteristics	Overall	SIB	Brachy	SMD
	Number (%)			
Number	162	81	81	
HPV status				0.164
Negative	21 (13.0)	10 (12.3)	11 (13.6)	
Positive	140 (86.4)	71 (87.7)	69 (85.2)	
Unknown	1 (0.6)	0 (0.0)	1 (1.2)	
Age ≥ median (62 years)	85 (52.5)	41 (50.6)	44 (54.3)	0.074
Tumour stage*				0.079
I-II	10 (6.2)	5 (6.2)	5 (6.2)	
III	18 (11.1)	8 (9.9)	10 (12.3)	
IV	134 (82.7)	68 (84.0)	66 (81.5)	
Tumour site				0.584
Base of tongue	90 (55.6)	34 (42.0)	56 (69.1)	
Tonsil	66 (40.7)	44 (54.3)	22 (27.2)	
Other	6 (3.7)	3 (3.7)	3 (3.7)	
Performance status at start of radiotherapy				< 0.001
0	144 (88.9)	72 (88.9)	72 (88.9)	
1	16 (9.9)	8 (9.9)	8 (9.9)	
2	2 (1.2)	1 (1.2)	1 (1.2)	
Male gender	127 (78.4)	63 (77.8)	64 (79.0)	0.030
Concurrent medical treatment				0.094
Cetuximab	80 (49.4)	41 (50.6)	39 (48.1)	
Cetuximab + Cisplatin	7 (4.3)	4 (4.9)	3 (3.7)	
Cisplatin	55 (34.0)	26 (32.1)	29 (35.8)	
None	20 (12.3)	10 (12.3)	10 (12.3)	
Induction chemotherapy	45 (27.8)	23 (28.4)	22 (27.2)	0.028
Medical treatment (concurrent or induction)	144 (88.9)	72 (88.9)	72 (88.9)	< 0.001
Cisplatin containing medical treatment	90 (55.6)	45 (55.6)	45 (55.6)	< 0.001

*SIB* Simultaneous integrated boost, *Brachy* Brachytherapy boost in combination with external beam radiotherapy, *HPV* Human papillomavirus, *SMD* Standardized mean difference

*Tumour stage according to the American Joint Committee on Cancer’s (AJCC) Cancer-Staging Manual, 7th edition

The analysis of acute and late grade ≥ 3 side effects showed no significant differences between the two different dose escalation techniques (Table 3). Six patients (2.5%) died from toxicity considered treatment related (Table 4). Out of these, two patients died of acute radiation toxicity (severe mucositis and infection) and one patient, who had received cisplatin, was pancytopenic and died of infection 3 weeks after completion of radiotherapy. The remaining 3 patients who were thought to have died due to treatment related toxicity, died of late complications: two from massive pharyngeal bleeding without evidence of recurrent disease (6 and 12 months after end of radiotherapy) and one patient from infection with origin in ORN of the mandible, 5 years after completion of radiotherapy.

**Table 3:** Comparison of grade ≥ 3 side effects between boost modalities

Side Effects	SIB	Brachytherapy	*p* value	Total
	Number (%)			
Skin				
1. Acute	28 (25.5)	29 (21.8)	0.742	57 (23.5)
2. Late	5 (4.5)	5 (3.8)		10 (4.1)
3. None	77 (70.0)	99 (74.4)		176 (72.4)
Osteoradionecrosis				
2. Late	9 (8.2)	11 (8.3)	1.000	20 (8.2)
3. None	101 (91.8)	122 (91.7)		223 (91.8)
Larynx				
1. Acute	0 (0.0)	0 (0.0)	1.000	0 (0.0)
2. Late	1 (0.9)	2 (1.5)		3 (1.2)
3. None	109 (99.1)	131 (98.5)		240 (98.8)
Salivary glands				
2. Late	8 (7.3)	9 (6.8)	1.000	17 (7.0)
3. None	102 (92.7)	124 (93.2)		226 (93.0)
Trismus				
1. Acute	1 (0.9)	0 (0.0)	0.545	1 (0.4)
2. Late	4 (3.6)	5 (3.8)		9 (3.7)
3. None	105 (95.5)	128 (96.2)		233 (95.9)
Mucosa				
1. Acute	71 (64.5)	83 (62.4)	0.817	154 (63.4)
2. Late	2 (1.8)	4 (3.0)		6 (2.5)
3. None	37 (33.6)	46 (34.6)		83 (34.2)
Dysphagia				
2. Late	16 (14.5)	24 (18.0)	0.577	40 (16.5)
3. None	94 (85.5)	109 (82.0)		203 (83.5)

*SIB* Simultaneous integrated boost, *Brachy* Brachytherapy boost in combination with external beam radiotherapy

**Table 4:** Grade 5 toxicity

Patient	Age (years)	Gender	Performance status at start of radiotherapy	Boost modality	Cause of death	Time of death (after end of radiotherapy)
1	79	Female	0	SIB	Acute radiation toxicity	3 weeks
2	81	Male	0	Brachytherapy	Massive pharyngeal bleeding	6 months
3	73	Male	2	SIB	Acute radiation toxicity	6 days
4	70	Male	1	SIB	Infection	3 weeks
5	59	Male	0	SIB	Massive pharyngeal bleeding	1 year
6	63	Male	0	SIB	ORN and infection	5 years

*SIB* Simultaneous integrated boost, *ORN* Osteoradionecrosis

## Discussion

The current analysis indicates that dose escalation in HNC results in similar survival and grade ≥ 3 side effects, whether the boost is delivered using external-beam radiotherapy only (SIB) or using a sequential brachytherapy boost. The literature comparing treatment results from SIB and brachytherapy boost in HNC is scarce. To our knowledge, the only reference is the single-centre report by Chen et al. [19] of their experience in treating base of tongue squamous cell carcinoma over three decades. During this period different treatment strategies were developed over time. In the first decade they mainly used external beam radiotherapy in combination with brachytherapy boost, in the second, conventional external beam radiotherapy and, in the third decade, IMRT as SIB or sequential boost. In this study they compared the different treatment strategies including the comparison of external beam radiotherapy combined with brachytherapy boost (median total dose of 75 Gy) and IMRT-based SIB (standard dose, 67.5 Gy in 30 fractions), and saw no significant differences in side effects but improved survival in the group that had received IMRT-based SIB (5-year OS 72% vs. 49%, *P* = 0.04). However, the authors point out that the differences in OS probably were influenced by the imbalance in the addition of chemotherapy, which was more common in the IMRT-based SIB group, and when corrected for this imbalance the significant differences in OS disappeared. However, the inconsistency of treatment strategies over time (addition of concurrent chemotherapy and better imaging technique which facilitates better target definition) makes the comparison difficult, and this study did not compare the two different techniques in the dose escalation setting. In contrast, our study compares patients treated during the same period and the patients in our two cohorts have received doses to boost volumes that are radiobiologically similar, which makes the comparison of treatment- and toxicity outcome more relevant.

In the current study there was an imbalance in the two treatment groups regarding tumour type. There were more patients treated for base of tongue cancer in the brachytherapy group and more tonsillar cancer in the SIB group. This is probably because the treatment modality was by clinician’s choice and the understanding that T1-2 tumours in the base of tongue have a poorer prognosis than tonsillar tumours of corresponding T stage [21, 22]. Consequently, among patients with T1 and T2 tumours, more patients with base of tongue cancer would be considered for dose escalated radiotherapy than patients with tonsillar cancer. However, regarding the imbalance in tumour location between the cohorts, no significant difference in survival related to tumour location was observed, indicating that the imbalance of tumour type in the treatment groups had little impact on the survival analysis comparing the different boost modalities.

The grade ≥ 3 side effects reported in the current study did not differ significantly between the group that had dose escalated radiotherapy by SIB or by brachytherapy boost. The incidence of ORN (8.2%) is in level with previously published data where ORN after brachytherapy in the head and neck area is reported in 2–9% of patients that are not previously irradiated [17, 26–31] and in 3–8.2% of patients treated with external beam radiotherapy to a standard curative dose [32–35]. The incidence of late mucosal ulcers and soft tissue necrosis in the current study (2.5%) is somewhat lower than described in previously published data. In patients who receive brachytherapy in the head and neck area (alone or in combination with external beam radiotherapy) the incidence of late mucosal ulcers is 4–14% [17, 26–31, 36] and in patients treated with dose escalated external beam radiotherapy the incidence of grade 4 mucosal ulcers has been reported as high as 20–23% [37, 38]. The lower incidence of late mucosal ulcers in our study might be due to our relatively moderate dose escalation, or to the retrospective design of our study and the risk of underreported side effects. But as our registry has prospectively gathered data on side effects, the latter is unlikely to be a major source of error. In contrast to late mucosal ulcers, the incidence of late severe dysphagia (16.5%) seems higher in the current study compared to previously published data. In the study by Chen et al., 6% of patients treated with brachytherapy in combination with external beam radiotherapy and/or surgery experienced severe dysphagia [17]. After external beam radiotherapy to standard curative dose, severe dysphagia is seen in 5–9% [39, 40]. A reason for our seemingly high rates of severe late dysphagia could be our longer follow-up of 6.1 years, compared to 4 years in the study by Chen et al., but also our higher total dose compared to the data from conventional external beam radiotherapy.

Lower grade side effects (grade 1 and 2) were not analysed in this study.

In the current study, 2.5% of patients were thought to have died due to treatment related toxicity. This is in line with other published data on lethal complications in 0–4% of cases after radiotherapy of oropharyngeal cancer [17, 41–47]. The causes of death were heterogenous, with both acute and late grade 5 toxicities. This makes it difficult to draw any statistical conclusions, but 5 out of 6 patients that were thought to have died due to treatment related toxicity were treated with dose escalated SIB and only one had brachytherapy. One explanation might be, selection bias since patients that are treated with brachytherapy have to be fit enough to undergo anaesthesia, while a more frail patient could still receive dose escalation by SIB. This is, however, not confirmed by any significant difference in PS at start of radiotherapy between the groups. But 2 of the patients with acute grade 5 toxicity had PS ≥ 1 at start of radiotherapy.

Although we saw no statistically significant differences in the pattern of recurrence between the two treatment groups, there are more regional recurrencies in the brachytherapy group (4, 3%) compared to the SIB group (0%). A review of treatment plans and diagnostic imaging showed that 3 out of 4 regional recurrencies were in-field recurrencies treated with 68 Gy at primary treatment and 1 recurrence was a marginal failure (ipsilateral lymph node metastasis in the cranial retropharyngeal region), mainly outside the elective volume of the primary treatment. The reason for more regional recurrencies in the brachytherapy group than in the SIB group is unclear.

The use of brachytherapy boost in oropharyngeal cancer has declined over time in our centre in favour of SIB, with or without dose escalation. This is consistent with the trend in some other centres [19]. There could be several different reasons for this, but one important factor is probably that the technological advancements have made it easier to use SIB, and brachytherapy could be considered more complex given the invasive surgical procedure, and it also requires physicians who master the craft of catheter implantation. The results of our study support the transition from brachytherapy to external beam radiotherapy and the use of SIB to achieve dose escalation in oropharyngeal cancer, as the outcomes are equivalent, and as SIB is less labour intense. But there might be clinical cases where the rapid dose fall-off of brachytherapy is considered superior and in that case our study also supports the use of brachytherapy. There are other indications for HNC brachytherapy that has not decreased over time in our centre, like cancer of the lip and cancer of the nasal vestibule, where brachytherapy in combination with external beam radiation still is the treatment of choice.

The side effects of dose escalation radiotherapy seen in this study will be further investigated in our next study, where we compare toxicity- and survival outcome in the dose escalation cohort with a matched cohort that received standard dose radiotherapy.

## Conclusions

In the current study we found no significant differences in OS, PFS or grade ≥ 3 side effects comparing simultaneous integrated boost and brachytherapy boost as alternative dose escalation modalities in the treatment of oropharyngeal cancer.

## Supplementary Information

**Additional file 1: Table S1**. Details on fractionation schedules. **Fig. S1**. Covariate balance between boost modality groups before and after propensity score match. **Fig. S2**. Overall survival (**a**) and progression-free survival (**b**) by tumour type.

## Data Availability

Research data are stored in an institutional repository and will be shared upon reasonable request to the corresponding author.

## Abbreviations

AJCC: American Joint Committee on Cancer
CT: Computed tomography
EORTC: European Organization for Research and Treatment of Cancer
EQD2: Equivalent dose in 2 Gy fractions
GTV: Gross tumour volume
GTVT: Gross tumour volume of the primary tumour
HDR: High dose rate brachytherapy
HNC: Head and neck cancer
HPV: Human papillomavirus
IMRT: Intensity modulated radiotherapy
LENT/SOMA: Late effects Normal Tissue Task Force Subjective, Objective, Management, and Analytic
MRI: Magnetic resonance imaging
ORN: Osteoradionecrosis
OS: Overall survival
PDR: Pulsed dose rate brachytherapy
PET: Positron emission tomography
PFS: Progression-free survival
PTV: Planning target volume
RTOG: Radiation Therapy Oncology Group
SIB: Simultaneous integrated boost
VMAT: Volumetric modulated arc therapy
