# Supplementary material for: Dose escalation in oropharyngeal cancer: a comparison of simultaneous integrated boost and brachytherapy boost
Source: Radiat Oncol. 2023 Apr 7;18:65. doi: 10.1186/s13014-023-02256-x (PMC10082532; doi:10.1186/s13014-023-02256-x)
Supplement: Supplementary file 1 — Additional file 1: Table S1. Details on fractionation schedules. Fig. S1. Covariate balance between boost modality groups before and after propensity score match. Fig. S2. Overall survival (a) and progression-free survival (b) by tumour type. [file 13014_2023_2256_MOESM1_ESM.docx]

**Table S1**. Details on fractionation schedules

| **Fractionation schedules** | **Number (percent)** | **Total dose in EQD2, α/β=3 Gy** | **Total dose in EQD2, α/β=10 Gy** |
| --- | --- | --- | --- |
| **External beam radiotherapy with SIB** | 110 (45) |  |  |
| 84 Gy, 2.4 Gy/fx, 35 fx | 2 (2) | 90.7 | 86.8 |
| 74.8 Gy, 2.2 Gy/fx, 34 fx | 99 (90) | 77.8 | 76.0 |
| 73.8 Gy, 2.05 Gy/fx, 36 fx | 1 (1) | 74.5 | 74.1 |
| 73.1 Gy, 2.15 Gy/fx, 34 fx | 6 (5) | 75.3 | 74.0 |
| 72.6 Gy, 2.2 Gy/fx, 33 fx | 2 (2) | 75.5 | 73.8 |
| **External beam radiotherapy 68 Gy, 2 Gy x 34 + brachytherapy boost** | 133 (55) |  |  |
| PDR 9.9 Gy, 0.66 Gy/fx, 15 fx | 35 (26) | 75.2 | 76.8 |
| PDR 11.8 Gy, 0.56 Gy/fx, 21 fx | 1 (1) | 70.7 | 76.0 |
| PDR 8.4 Gy, 0.56 Gy/fx, 15 fx | 90 (67) | 74.0 | 75.4 |
| HDR 24 Gy, 3 Gy/fx, 8 fx | 1 (1) | 96.8 | 94 |
| HDR 12 Gy, 3 Gy/fx, 4 fx | 1 (1) | 82.4 | 81.0 |
| HDR 10 Gy, 2.5 Gy/fx, 4 fx | 1 (1) | 79.0 | 78.4 |
| HDR 9 Gy, 3 Gy/fx, 3 fx | 3 (2) | 78.8 | 77.8 |
| HDR 7.5 Gy, 2.5 Gy/fx, 3 fx | 1 (1) | 76.3 | 75.8 |
| **External beam radiotherapy of the neck prior to brachytherapy boost** | 133 (55) |  |  |
| 46 Gy, 2 Gy/fx, 23 fx | 117 (89) | 46.0 | 46.0 |
| 51.68 Gy, 1.52 Gy/fx, 34 fx | 10 (8) | 46.7 | 49.6 |
| 68 Gy, 2 Gy/fx, 34 fx | 4 (3) | 68.0 | 68.0 |
| **External beam radiotherapy 74.8 Gy, 2.2 Gy x 34 + brachytherapy boost** | 1 (0) |  |  |
| PDR 8.4 Gy, 0.56 Gy/fx, 15 fx | 1 | 83.8 | 83.4 |

EQD2 - equivalent dose in 2 Gy fractions, SIB – simultaneous integrated boost, fx – fraction, PDR - pulsed dose rate brachytherapy, HDR - high dose rate brachytherapy

**Figure S1**. Covariate balance between boost modality groups before and after propensity score match.

**Figure S2**. Overall survival (a) and progression-free survival (b) by tumour type. BoT – base of tongue
